# Analysis of a genetic region affecting mouse body weight

**DOI:** 10.1152/physiolgenomics.00137.2022

**Published:** 2023-01-30

**Authors:** Connie L. K. Leung, Subashini Karunakaran, Michael G. Atser, Leyla Innala, Xiaoke Hu, Victor Viau, James D. Johnson, Susanne M. Clee

**Affiliations:** Department of Cellular and Physiological Sciences, Life Sciences Institute, University of British Columbia, Vancouver, British Columbia, Canada

**Keywords:** BTBR, genetics, mice, pyruvate dehydrogenase kinase 1, triglycerides

## Abstract

Genetic factors affect an individual’s risk of developing obesity, but in most cases each genetic variant has a small effect. Discovery of genes that regulate obesity may provide clues about its underlying biological processes and point to new ways the disease can be treated. Preclinical animal models facilitate genetic discovery in obesity because environmental factors can be better controlled compared with the human population. We studied inbred mouse strains to identify novel genes affecting obesity and glucose metabolism. BTBR T+ *Itpr3^tf^*/J (BTBR) mice are fatter and more glucose intolerant than C57BL/6J (B6) mice. Prior genetic studies of these strains identified an obesity locus on chromosome 2. Using congenic mice, we found that obesity was affected by a ∼316 kb region, with only two known genes, pyruvate dehydrogenase kinase 1 (*Pdk1*) and integrin α 6 (*Itga6*). Both genes had mutations affecting their amino acid sequence and reducing mRNA levels. Both genes have known functions that could modulate obesity, lipid metabolism, insulin secretion, and/or glucose homeostasis. We hypothesized that genetic variation in or near *Pdk1* or *Itga6* causing reduced *Pdk1* and *Itga6* expression would promote obesity and impaired glucose tolerance. We used knockout mice lacking *Pdk1* or *Itga6* fed an obesigenic diet to test this hypothesis. Under the conditions we studied, we were unable to detect an individual contribution of either *Pdk1* or *Itga6* to body weight. During our studies, with conditions outside our control, we were unable to reproduce some of our previous body weight data. However, we identified a previously unknown role for *Pdk1* in cardiac cholesterol metabolism providing the basis for future investigations. The studies described in this paper highlight the importance and the challenge using physiological outcomes to study obesity genes in mice.

## INTRODUCTION

Obesity and type 2 diabetes are complex diseases that share common risk factors. These metabolic diseases share inter-related traits such as increased BMI, adiposity, hyperinsulinemia, insulin resistance, and mitochondrial dysfunction. The discovery of genetic factors affecting these traits in humans has been productive but challenging, due to the relatively small effects of many genetic variants, and the effects of interindividual variation in environmental and lifestyle factors that can impact the phenotypic expression of these genetic changes. Genome-wide association studies (GWAS), and advances in sequencing technology have allowed the identification of loci that affect obesity susceptibility in the general population. However, the known common variants affecting the general population, added together, have modest effects on body weight. For example, GWAS data from a cohort of 694,649 samples found 346 loci associated with BMI had relatively small individual effect sizes, and the combined loci only accounted for ∼3.9% of the variation in BMI (1). In addition to genetic factors, lifestyle factors such as stress (2, 3), availability to and access to healthy food, or socioeconomic status (4) have been associated with obesity. This complexity confounds the discovery of obesity genes in humans.

As an alternative approach to overcome some of these challenges, we have capitalized on the natural genetic differences that occur between inbred mouse strains, and the strong evolutionary conservation of obesity mechanisms across species. The BTBR T+ *Itpr3^tf^*/J (BTBR) mouse strain has a high propensity for obesity. Prior studies have shown that this strain harbors alleles that promote metabolic disease compared with the C57BL/6J (B6) strain (5–8). Genetic studies identified several quantitative trait loci (QTLs) that affect metabolic disease traits in these strains (7, 8), and genes affecting these traits have been identified (9, 10). Additional insights about factors affecting metabolic disease in these strains have come from multiple “omic” studies and their integration with genetics (11–19).

One quantitative trait locus (QTL) identified using these strains is Modifier of obese 1 (*Moo1*) (7). At this locus, BTBR alleles were associated with increased body weight. We have previously localized this QTL to a ∼6 Mb region of chromosome 2 and have shown that it is associated with multiple metabolic traits (7, 20). Here, we report that *Moo1* comprises at least two QTLs, localized the major effect to a 316 kb region of chromosome 2, described the environmental modification of this locus by stress, as well as insights into lipid metabolism when PDK1 (pyruvate dehydrogenase kinase 1) levels are reduced.

## METHODS

### Animals

Animals were housed in ventilated microisolator cages in environmentally controlled facilities with 12-h light:dark cycles, unless otherwise noted. All mice were generated from crosses between heterozygotes (HETs) such that littermates were used as controls. Congenic mice with B6 regions of chromosome 2 replacing those of the BTBR background strain were generated from an in-house breeding colony. Subcongenic strains were created from recombinants of the Moo1C strain previously described (20). A heterozygous breeder pair of *Pdk1*^tm1.1(KOMP)Vlcg^ knockout (KO), global knockout mice, were obtained from the Knockout Mouse Project (KOMP), then bred in-house. In these mice, the *Pdk1* coding region is replaced by LacZ. These mice were originally on the C57BL/6N background but were backcrossed to C57BL/6J as part of colony expansion and maintenance. Because prior studies showed no effect of this locus in females, experiments used exclusively male animals (personal communication from Dr. Susanne Clee). Floxed *Itga6* mice were obtained from the laboratory of Dr. Elisabeth Georges-Labouesse, where the *Itga6* gene was flanked by two loxP sites. When Cre recombinase is expressed, the 3′ end of the transmembrane domain and the cytoplasmic A and B exons of *Itga6* are deleted (21, 22). The mice were on a mixed C57BL/6J x 129 Sv background and were re-derived into CDM (with 2 additional backcrosses to C57BL/6J), but the remaining 129 Sv genetic contribution is unknown. Heterozygous *Itga6* floxed mice were bred with mice heterozygous for cre recombinase driven by the ubiquitously expressing β-actin promoter (B6N.FVB-Tmem163Tg(ACTB-cre)2Mrt/CjDswJ mice; Stock Number 019099, The Jackson Laboratory; breeding animals were received from an in-house colony as a kind gift from the laboratory of Dr. Timothy Kieffer) to knockout *Itga6* in all cells from early in development. The goal was to obtain heterozygous *Itga6* KO (HET KO) animals along with littermate heterozygous floxed (FLOX), cre-expressing (CRE) and wild-type (WT) animals. Homozygous *Itga6* KO animals die soon after birth from severe epithelial blistering (21), thus we used heterozygous Itga6 KO mice in our studies.

Over the course of these studies, mice were housed in multiple facilities and rooms within these facilities, Center for Disease Modelling (CDM) *room 1*, *room 2*, and *room 3* and Wesbrook Mouse Facility. The majority of the studies were completed within the CDM at the University of British Columbia (UBC). Analysis of the subcongenic strains was performed concurrently with mice housed in *room 1* in CDM. However, CDM underwent reorganization, and the mice were moved to CDM *room 2*. The stress study was conducted in CDM *room 2*. CDM then underwent construction and thus when CDM reopened, repeat analysis of Moo1V strain concurrent with *Itga6* KO and repeat analysis *Pdk1* KO in CDM *room 3* was conducted.

At weaning, all experimental animals were placed on a diet high in fat with 20% kcal sucrose, and 60% kcal fat (D12492i, Research Diets). Mice were weighed weekly, at a standardized time of day. At the specified times, mice were euthanized by an overdose of isoflurane anesthesia in accordance with the Animal Care guidelines. At this time a cardiac blood sample was withdrawn, and tissues were rapidly harvested, weighed, and flash frozen. Blood samples were kept on ice, then plasma was separated by centrifugation at 4°C at 10,000 rpm for 8 min. Tissue and plasma samples were stored at −80°C until analysis. All procedures were approved by the University of British Columbia Animal Care and Use Committee and were performed in accordance with Canadian Council on Animal Care Guidelines (Protocol No. A19-0267).

### Genotyping

DNA was extracted from ear notches using a commercially available kit (Puregene, Qiagen). Congenic mice were genotyped at the first and last markers shown to be B6 within the congenic insert. Recombinants were confirmed, then additional markers were genotyped to fine-map the recombination. Genotyping of single-nucleotide polymorphism (SNP) markers was performed using high-resolution melt curve analysis (Qiagen kit) using the Rotor-gene Q thermocycler (Qiagen). Microsatellite markers were genotyped by PCR amplification and resolution on polyacrylamide gels, as described (23). *Pdk1* KO mice were genotyped by PCR amplification of a 195 bp band for the WT allele and a 639 bp band for the KO allele as recommended by KOMP (23). Genotyping of *Itga6* mutant mice was conducted with primers flanking the region of the loxP sites to amplify a fragment of 120 bp in WT animals and, 150 bp when the loxP site is present. Genotyping for the presence of cre recombinase was performed using a set of primers that amplify across the deleted region were used to amplify a fragment of 600 bp, as described (24). Primer sequences are provided in Supplemental Table S1. All genotyping reactions contained DNA samples known to be of each genotype as controls, along with a no template control.

### Gene Expression Analysis

Tissue samples were processed, and RNA was extracted using E.Z.N.A total RNA extraction kit (Omega Bio-tek, Norcross, GA), after which cDNA was generated using the RevertAid First Strand cDNA Kit (Thermo Fisher Scientific, Cat. No. K1631, Waltham, MA). Gene expression was measured using real-time qPCR with SYBR green. β-actin (*Actb*) was used as a reference gene as it more consistently amplified in the same manner across different experimental groups and tissues compared with glyceraldehyde 3-phosphate dehydrogenase (*Gapdh*) or cyclophilin (*Ppib*). A single product was verified using melt curve analysis. Cycle threshold value (Ct) was subtracted from the control gene Ct value, to give the ΔCt value (dCt). Then ΔΔCt values (ddCt) for each mouse were calculated by subtracting each dCt value from the average dCt of the control group. No reverse transcriptase (no RT) and no template control (water) were used as negative controls and were determined for each gene and tissue assessed. Primer sequences are provided in Supplemental Table S2.

### Body Weight and Blood Sampling

Mice were weighed weekly at a standardized time of day from weaning at 3 wk until 10 wk of age. To determine changes in body composition, dual-energy X-ray absorptiometry (DEXA) analysis was performed using a PIXImus Mouse Densitometer (Inside Outside Sales, Madison, WI). Body weight, body length, and cardiac blood samples were also obtained at the time of euthanasia.

### Stress Study

To directly assess the interaction between stress and genotype on obesity, we used chronic restraint stress, a well-established model of stress in rodents (25). At 10 wk of age, the mice were singly housed for the duration of the study. At 11 wk of age, restraint stress was induced in half the mice by placing the mice in a plexiglass restrainer for 90 min from 9:00 AM to 10:30 AM daily, for 7 days. The plexiglass restrainer confined the mice into a small space, with no room to move, but it did not specifically immobilize them. The control mice were singly housed during the week of stress but were not placed in the restrainers; however, all the other hormone and metabolic measurements were performed concurrently with the stress group. Age-matched littermates were used in both the control and stressed groups. To assess food intake during the experiment, mice were given a pre-weighed amount of food at the start of the study. The weight of the food remaining at the end of the study was determined again to calculate the total food intake during the 1 wk of restraint stress. There were no visible crumbs of food in the cages when the food weight was measured. Corticosterone levels were measured in plasma from the cardiac bleeds of the mice using a Corticosterone Double Antibody radioimmunoassay kit (MP Biomedicals, Solon, OH) as previously described (26). Thirty-six hours after the last stress induction, the mice were euthanized and a standard set of tissue samples for all the mice were collected, including brain, heart, stomach, intestines, spleen, fat deposits [epididymal, renal, mesenteric, brown adipose tissue (BAT)], soleus muscle, and testes. These tissues were weighed and then flash frozen on dry ice as soon as they were collected. Immediately after euthanasia, before tissue collection, cardiac blood samples were collected and placed into microcentrifuge tubes containing 6 µL of 25 mM EDTA and kept on ice until plasma separation. All plasma samples were separated by centrifugation at 4°C at 10,000 rpm for 8 min, and all tissue and plasma samples were stored at −80°C until analysis.

### Glucose and Triglycerides

Metabolite levels were measured in plasma from the cardiac blood samples collected from mice at the time of euthanasia. The mice were fasted for 4 h before euthanization. Glucose levels were measured with a colorimetric assay as described earlier (Autokit Glucose Cat. No. 997-03001, Wako Diagnostics, Richmond, VA). Insulin was measured by ELISA (Mouse Ultrasensitive Insulin ELISA, Cat. No. 80-INSMSU-E01, Alpco, Salem, NH). Plasma β-hydroxybutyrate levels (Beta Hydroxybutyrate Assay kit, Cat. No. ab83390, Abcam, Cambridge, MA), cholesterol (Cholesterol-SL Assay, Cat. No. 234-60 Sekisui Diagnostics, PEI, Canada), triglyceride and glycerol (Triglyceride-SL Assay, Cat. No. 236-60, Sekisui Diagnostics, PEI, Canada), and pyruvate (Pyruvate Assay Kit, Cat. No. MAK071, Sigma-Aldrich, Oakville, ON, Canada) were measured using colorimetric assays according to the manufacturers’ directions. The products of the colorimetric assay were measured spectrophotometrically at 505 nm for glucose and cholesterol, at 520 nm for triglycerides, at 450 nm for insulin and β-hydroxybutyrate, and at 570 nm for pyruvate using an Infinite M1000 microplate reader (Tecan, Durham, NC).

Tissue cholesterol and triglyceride levels were measured in liver and heart samples (27). Tissues (∼100 mg) were homogenized using a tissue homogenizer in 3 mL of chloroform and methanol (2:1), and extracted using 1.5 mL of ice-cold water and a second time with 750 µL of ice-cold water. A third of the measured organic layer from the liver extraction was dried with 30 µL of Thesit (hydroxypolyethoxydodecane) neat (Sigma-Aldrich, Oakville, ON, Canada) under nitrogen gas. The volume used for dehydration was measured precisely after the aliquot, as to not disturb the organic layer. For the heart samples, the entire organic layer was dried. Standards with cholesterol (Wako Diagnostics, Richmond, VA) and triolein (1:250 uL) (Sigma-Aldrich, Oakville, ON, Canada) were dried under nitrogen gas. Once dried, the samples were mixed with 300 µL of water and incubated at 37°C with vigorous vortexing twice partway through the 30-min incubation. Samples were stored at 4°C until analysis. Before the analysis, samples were brought up to room temperature and diluted with 10% Thesit as needed, as determined by first testing the undiluted samples and then repeating the assay on samples that were not on the standard curve (28).

Tissue cholesterol was measured using a colorimetric assay (Cholesterol E, Cat. No. 439-17501, Wako Diagnostics, Richmond, VA). The cholesterol reagents were reconstituted according to the manufacturer’s instructions. In a flat bottom 96-well plate (Microtest plate 96 well, Cat. No. 82.158, Sarstedt, Nümbrecht, Germany), 20 µL of extracted lipids and standards were added, then 200 µL of cholesterol E reagent (Cholesterol E, Cat. No. 439-17501, Wako Diagnostics, Richmond, VA) was added and incubated at room temperature for 5 min. The products of the colorimetric assay were measured spectrophotometrically at 590 nm using an Infinite M1000 microplate reader (Tecan, Durham, NC). The values were expressed per amount of starting protein and accounted for the dried volume.

Tissue triglyceride levels were measured using an enzymatic triglyceride assay (Glycerol Reagent, Cat. No. F6428, Triglyceride Reagent, Cat. No. T2449, Sigma-Aldrich, Oakville, ON, Canada). In a flat bottom 96-well plate, (as described earlier), 5 µL of extracted lipids and standards and 200 µL Glycerol Reagent (Glycerol Reagent, Cat. No. F6428, Sigma-Aldrich, Oakville, ON, Canada) were added and first measured to obtain a reading for glycerol at 540 nm. Then 50 µL of Triglyceride Reagent (Triglyceride Reagent, Cat. No. T2449, Sigma-Aldrich, Oakville, ON, Canada), which contains lipase to release glycerol from the fatty acids, was added to each of the samples. The absorbance values of the plate were measured at 540 nm using an Infinite M1000 microplate reader (Tecan, Durham, NC). The samples were incubated for 5 min at 37°C before each absorbance reading. The last absorbance read was then subtracted from the initial plate absorbance to obtain the final absorbance due to glycerol from triglycerides.

To examine triglyceride synthesis and secretion in *Pdk1* KO mice, a standard lipogenesis test (triglyceride synthesis and secretion) was conducted using poloxamer 407 (29) at 12 wk of age. Poloxamer 407 is a chemical that blocks triglyceride breakdown and clearance from the blood by inhibiting lipoprotein lipase (29). Thus, during fasting, increasing plasma triglyceride concentrations result from increased triglyceride synthesis and secretion. The mice were fasted for 4 h before testing. Blood was collected at *time 0*, and *30*, *60*, *120*, *240*, and *360* min posttreatment of injection with poloxamer 407 (1 g/kg ip). Saphenous blood was collected into tubes containing 6 µL of 25 mM EDTA and kept on ice until plasma separation. All plasma samples were separated by centrifugation at 4°C at 10,000 rpm for 8 min. Plasma was stored at −80°C until further analysis.

To assess lipid clearance from circulation in *Pdk1* KO mice, an oral lipid tolerance test was performed at 14 wk of age. Mice were fasted overnight (5:00 PM to 9:00 AM). At the time food was removed, the mice were placed in clean cages with water before the lipid tolerance tests and kept in these cages throughout the testing to ensure that the triglycerides in the plasma were from the oral gavage instead of from food crumbs. For the lipid tolerance, saphenous blood was collected at *time 0*, *30*, *60*, *120*, *240*, and *360* min post oral gavage of olive oil (300 µL) (30). Saphenous blood was collected into tubes containing 6 µL of 25 mM EDTA and kept on ice until plasma separation. All plasma samples were separated by centrifugation at 4°C at 10,000 rpm for 8 min and stored at −80°C until further analysis.

### Western Blotting

Western blotting was performed to analyze the protein levels of PDK1. Hearts were isolated from mice as previously described, flash frozen in liquid nitrogen, and ground with a liquid nitrogen-chilled mortar and pestle. A portion of the ground tissue was put in Radioimmunoprecipitation assay lysis buffer: 150 mM NaCl, 1% Nonidet P-40, 0.5% sodium deoxycholate, 0.1% sodium dodecyl sulfate (SDS), 50 mM Tris, pH 7.4, 2 mM EGTA, 2 mM Na_3_VO_4_, and 2 mM NaF supplemented with complete mini protease inhibitor cocktail (Roche, Laval, QC), then frozen at −80°C and thawed. Protein concentration in the lysates were measured using a Pierce bicinchoninic acid (BCA) protein assay kit (Cat. No. 23225, Thermo Fisher Scientific). Protein lysates were incubated in Laemmli loading buffer (Thermo, J61337AC) containing dithiothreitol at 95°C for 5 min. Fifteen micrograms of each sample were resolved on a 12% SDS-PAGE. Proteins were then transferred to PVDF membranes (Bio-Rad, CA) and probed with antibodies against PDK1 (1:2,500, Cat. No. ab202468, Abcam), β-tubulin (1:2,000, Cat. No. T8328, Sigma). The signals were detected by secondary horseradish peroxidase (HRP)-conjugated antibodies (1:10,000, Anti-mouse, Cat. No. 7076; Anti-rabbit, Cat. No. 7074; Cell Signaling Technologies) and Pierce ECL Western Blotting Substrate (Thermo Fisher Scientific).

### Statistical Analyses

Statistical analyses, including two-way ANOVA and Student’s *t* test, were performed using GraphPad Prism Software (v.6.0, La Jolla, CA). A *P* value of less than 0.05 was considered significant.

## RESULTS

### Localization of *Moo1*

**Figure 1.**
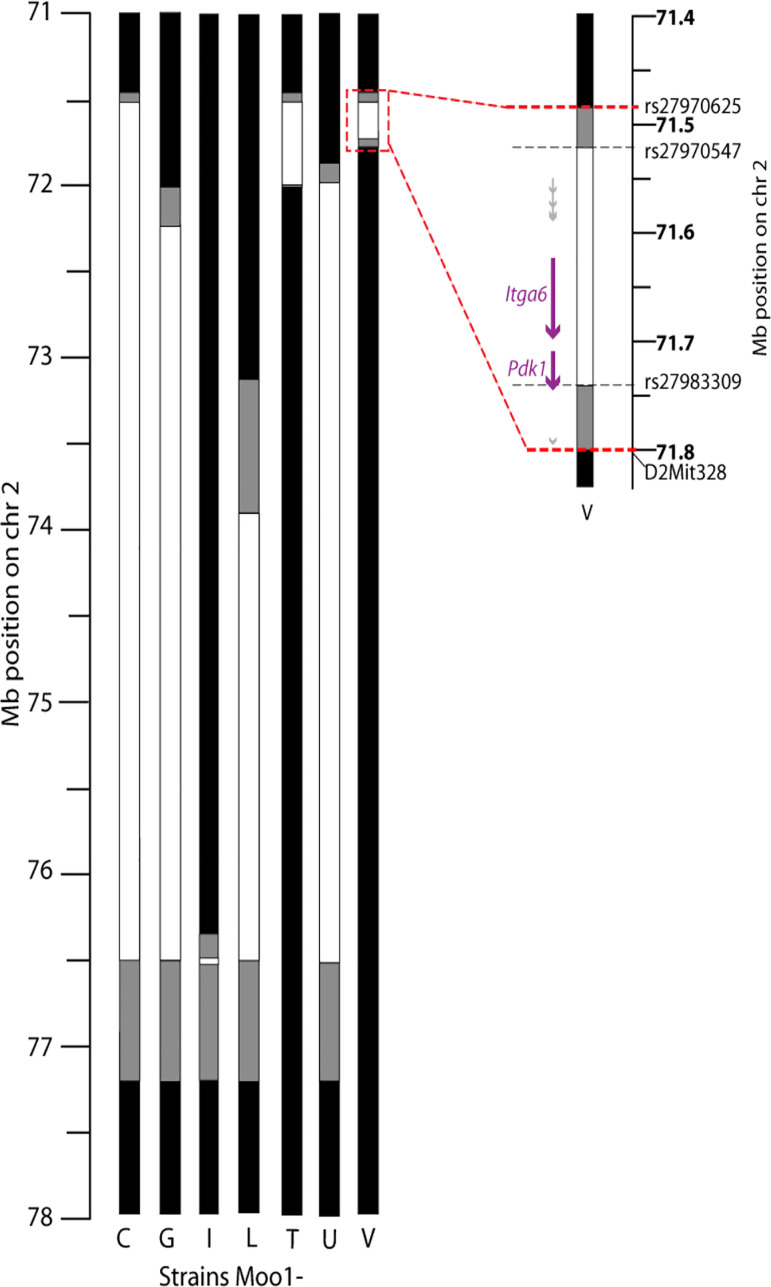
Modifier of obese 1 (*Moo1*) subcongenic strains. Subcongenic strains with B6 congenic inserts in a BTBR genomic background, drawn to scale. Positions on chromosome 2 are from the mm9 genome assembly. The markers defining the boundaries of the Moo1V strain are shown on the zoomed version. White boxes represent the B6 congenic insert, gray boxes are undetermined genotype between the last markers tested as B6 and the first marker tested as BTBR, black = BTBR region as throughout the rest of the genome. On the zoomed version, the location of the known genes (*Itga6*, *Pdk1*; purple) and predicted genes (from GENCODE VM20; gray). The upper gray line includes five predicted genes: *Platr26, Gm13647, Gm13663, Gm17250, Gm13662*; the gray arrow at the bottom of the region is *Gm13746*. None of these predicted genes are shown to have orthologues in other species, including rat which is very closely related to mouse.

Previous studies in Dr. Alan Attie’s laboratory at the University of Wisconsin had mapped a region that causes an increase in body weight in BTBR mice compared with B6 mice on the *ob/ob* background, despite the extreme hyperplasia of the mice without functional leptin, to chromosome 2. Fine mapping was done using congenic mice to localize a region on chromosome 2 that causes an increase in body weight when BTBR alleles are inherited (7). The chromosome 2 region found in the ob/ob mouse background was also found to affect high-fat diet (HFD)-induced obesity. A recent independent genetic cross also showed a significant linkage peak in this region (Supplemental Fig. S1) (16, 17). We have previously described the localization of *Moo1* to a ∼6 Mb region of chromosome 2 contained within a congenic strain, Moo1C (20). To further genetically localize *Moo1*, we created a panel of subcongenic strains from recombinants of the Moo1C strain (Fig. 1).

**Figure 2.**
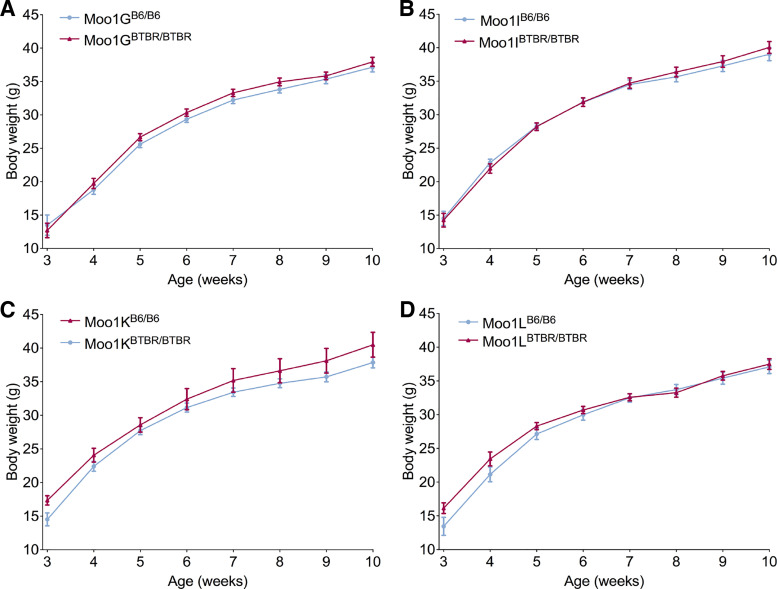
Body weight analysis of *Modifier of obese 1* (*Moo1*) subcongenic strains. Male mice were fed the same high-fat diet (HFD) housed in Center for Disease Modelling (CDM) room 1 with the Moo1C and Moo1V mice, and weighed weekly. Neither Moo1G strain mice (*A*), Moo1I strain mice (*B*), Moo1K strain mice (*C*), nor Moo1L strain mice (*D*) had significant differences in body weight between genotypes. Data are means ± SE analyzed by two-way ANOVA. ns, not significant.

Before these mice were moved to the Clee laboratory at UBC, the congenic mice were backcrossed to remove the ob/ob allele background. We assessed body weights weekly in mice from each strain, as in our prior studies in the genetic screens (Fig. 2) (20). These studies found that at least two independent loci within the Moo1C region contributed to the QTL, even without the ob/ob allele background. A major locus was localized to a 316 kb region in the Moo1V strain, and has been termed *Moo1a*. A second locus having a smaller effect was localized telomeric to this region in the nonoverlapping Moo1G strain and has been termed *Moo1b*. For the studies described in this paper, we focused on Moo1V strain (*Moo1a*).

**Figure 3.**
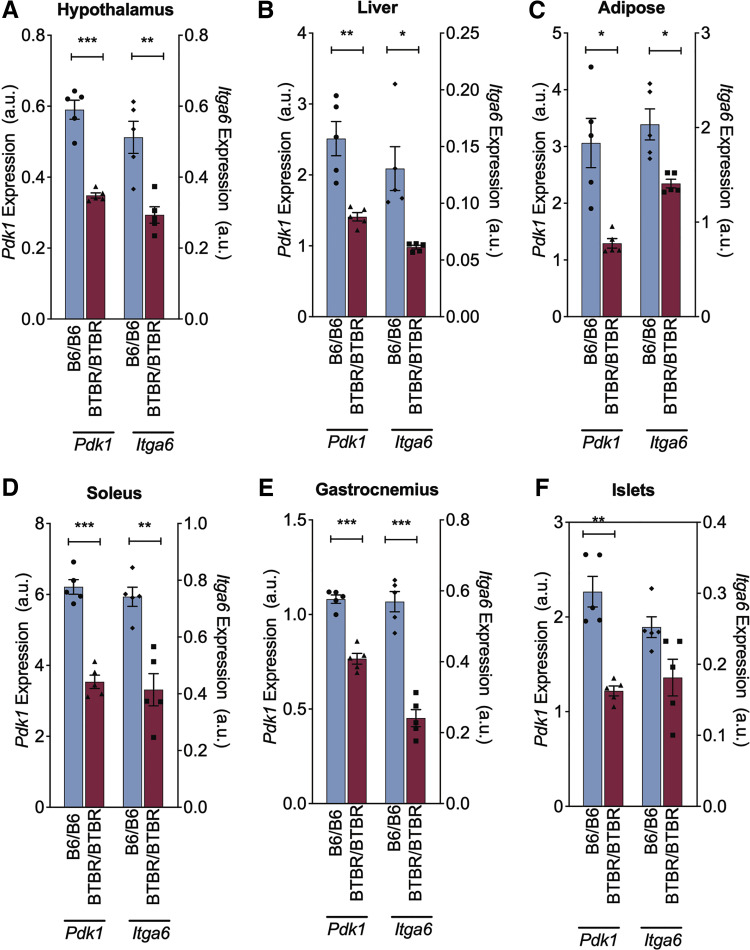
Gene expression of *Pdk1* and *Itga6* is reduced in BTBR and B6 parental strains. Gene expression in hypothalamus (*A*), liver (*B*), adipose tissue from epididymal fat pad (*C*), soleus muscle (*D*), gastrocnemius muscle (*E*), and islets (*F*) from 4-wk-old B6 (red, *n* = 5) or BTBR (blue, *n* = 5) mice fed chow at University of Wisconsin-Madison (UW). Data are raw microarray expression values, obtained from diabetes.wisc.edu, and are shown as means ± SE. Statistics were performed by Student’s *T* test (*P < 0.05, **P < 0.01, ***P < 0.001).

To identify genetic differences between the strains, we designed primers flanking each exon and spanning ∼2 kb of promoter for *Itga6* and *Pdk1*, and performed Sanger sequencing. Numerous differences were identified (Table 1). Notably, within the coding region of *Itga6* was a nonsynonymous change that results in a leucine in B6 mice and a valine in BTBR (now rs13464795). Notably, valine is present in many other species, suggesting it may be the ancestral allele (Supplemental Fig. S2). Within the coding region of *Pdk1* was a 6 bp insertion/deletion (indel) that results in the presence of an alanine and serine at amino acids 27 and 28 in B6 mice, but their absence in the BTBR strain (now rs222188523). Interestingly, the N-terminal region of PDK1 varies in length across species (Supplemental Fig. S3), so the functional importance of this change is unclear, however in the mouse strains with sequence data available, these two amino acids appear only in B6 (Supplemental Figs. S3 and S4). In both genes, we also identified many variants in noncoding regions, synonymous variants, and variants in the untranslated regions (Table 1). Since this work was performed, many mouse strains in addition to B6 have been sequenced, including BTBR. These data, retrieved from the Mouse Phenome Database, list a total of 3,416 variants (SNPs and indels) differing between the B6 and BTBR strains in the 316 kb region of the Moo1V strain. Because we identified many noncoding variants around *Itga6* and *Pdk1*, we examined whether these may affect their expression in multiple tissues. These studies showed that Moo1^BTBR/BTBR^ mice have a ∼50% reduction in gene expression of *Pdk1* and *Itga6* in most tissues compared with Moo1^B6/B6^ mice (Fig. 3). These data are consistent with a recent genetic analysis of these strains that identified cis-eQTLs (16, 17) for both *Pdk1* and *Itga6* (Supplemental Fig. S1). Thus, we considered both *Pdk1* and *Itga6* as positional candidate genes.

**Table 1.** Sequence differences between B6 and BTBR in Pdk1 and Itga6

		In mRNA			
Gene	*n* in 2 kb Promoter	Nonsynonymous	Synonymous, *n*	Untranslated Region	Intronic, *n*
*Pdk1*	10	Del Ala, Ser27,28	Leu96 Asp99 Asp426	83	23
*Itga6*	11	Leu975Val	Arg91 Val119 Leu157 Thr243 Val290 Ala299 Ile333 Leu522 Leu952	1	47

Data showing the number of changes in BTBR mice in each gene, by location, obtained by Sanger sequencing performed by the Clee Lab. The first ∼2 kb of the promoter was sequenced, and only short segments of each intron immediately flanking the exons were sequenced. There is a 6 nucleotide deletion resulting in the deletion of an alanine and serine in PDK1. A mutation that results in the change of a leucine to valine in ITGA6 was also found. The number of variants in the promoter, introns, and mRNA (that were nonsynonymous, synonymous, or in the untranslated region) is shown. Data obtained subsequently from the Jax Mouse Phenome Database website (https://phenome.jax.org/projects/Sanger4) from large scale genotyping confirmed these variants and identified 2,922 sequence variants between B6 and BTBR in the entire 316 kb region.

### Environmental and Stress Modulation of *Moo1*

**Figure 4.**
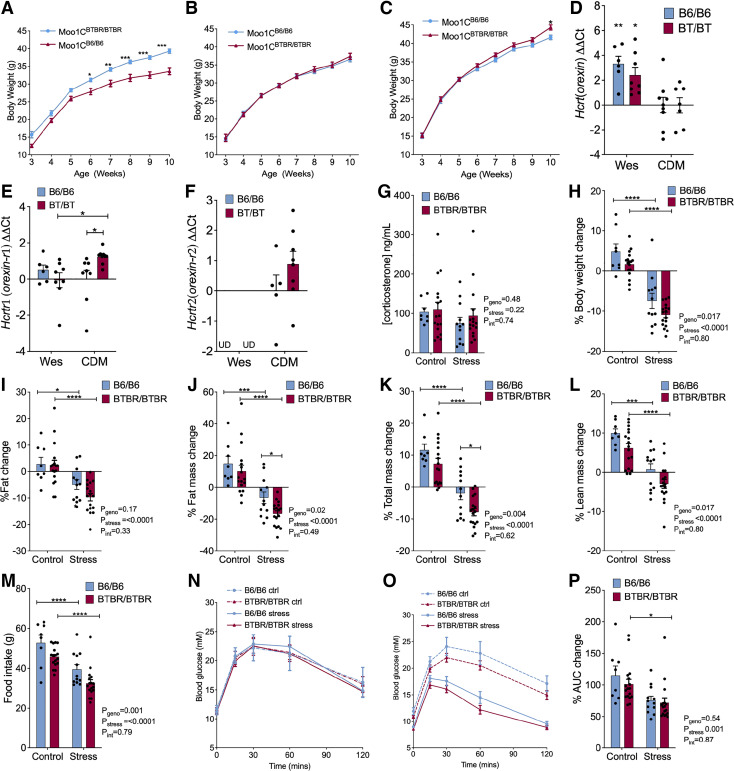
Environmental and stress modulation of the body weight phenotype. Mice were moved from the University of Wisconsin-Madison (UW) to the University of British Columbia (UBC-Wesb) and into a second facility at UBC (UBC-CDM). *Modifier of obese 1* (*Moo1*)C growth curves in UW (*A*), Wesbrook (*B*), and Center for Disease Modelling (CDM) facilities (*C*). Orexin (*Hcrt*) (*D*), Orexin receptor 1 (*Hcrtr1*) (*E*), and Orexin receptor 2 (*Hcrtr2*) (*F*) expression levels in the brain were assessed in Moo1C congenic mice from the Wesbrook (Wesb) facility compared with the CDM facility. Data are ΔΔCt (ddCt) values relative to Moo1C^B6/B6^ mice in CDM. *n* = 6–9. *G*: corticosterone levels in cardiac blood samples of Moo1V strain mice at 12 wk of age did not differ. Mice corticosterone levels were not significantly different in all four groups. Mice were housed in CDM *room 2* and were fed a high-fat diet (HFD). *H*: body weight was assessed in 12 wk mice 24 h before and 24 h after the last stress treatments. Moo1V^B6/B6^(B6/B6) control *n* = 8, stress *n* = 12; Moo1V^BTBR/BTBR^ (BTBR/BTBR) stress and control group *n* = 17/group. Body composition was assessed by dual-energy X-ray absorptiometry (DEXA) before and after the one week experiment. Percent change in percent body fat (*I*), fat mass (*J*), total mass (*K*), and lean mass (*L*). Moo1V^B6/B6^ control *n* = 8, Moo1V^B6/B6^ stress *n* = 12, Moo1V^BTBR/BTBR^ stress *n* = 17, and Moo1V^BTBR/BTBR^ control group *n* = 17. *M*: food intake was assessed in each group during the week of stress, *day 7* food weight was subtracted from that given on *day 1* of the experiment. Moo1V^B6/B6^ control *n* = 8, Moo1V^B6/B6^ stress *n* = 12, Moo1V^BTBR/BTBR^ stress *n* = 17 and Moo1V^BTBR/BTBR^ control group *n* = 17. Glucose tolerance tests (GTTs) were conducted both 24 h before and after the 1 wk of stress treatment and at the same time in nonstressed controls. Glucose tolerance in mice before (*N*) and after (*O*) the stress treatment were measured and area under the curve (AUC) (*P*) was calculated as area under the curve to *t* = 0. Moo1V^B6/B6^ (B6/B6) control *n* = 8, Moo1V^B6/B6^ stress *n* = 12, Moo1V^BTBR/BTBR^ (BTBR/BTBR) stress *n* = 17, and Moo1V^BTBR/BTBR^ control group *n* = 17. Data are shown as mean percent change in each group ± SE and were analyzed by two-way ANOVA with stress treatment and genotype as the factors (shown below each graph, along with the *P* value for their interaction). Bonferroni pairwise comparisons between groups that were significantly different are shown (*P < 0.05, **P < 0.01, ***P < 0.001, ****P < 0.0001) or ns for not significant.

During our studies of the Moo1C strain, the mice were relocated to a new university (UBC) and multiple housing locations (see, methods). The effect of *Moo1* on body weight in these strains varied between locations, even becoming undetectable in one location (Fig. 4, *A*–*C*). Subsequently, similar environmental effects were observed for the Moo1V strain housed in different rooms in the same facility (Supplemental Fig. S5). Collectively, these observations indicate that gene-environment interactions play a strong role in the penetrance of this phenotype.

One notable difference between the housing locations was the noise and potential disturbances to the mice due to adjacent construction. We assessed the expression of genes involved in pathways by which stress is known to affect food intake in the brains from Moo1C mice housed at the two UBC facilities, CDM and Wesbrook. Orexin mRNA levels were four- to eightfold higher in mice housed in the Wesbrook facility compared with the mice housed in the CDM, suggesting that differences between the facilities affected this pathway (Fig. 4, *D–F*).

To directly test whether stress modifies the effect of *Moo1* on body weight, we conducted controlled stress experiments to identify stress effects on body weight and composition, food intake, and glucose tolerance. To determine the degree to which mice were stressed due to their placement in the restrainers, circulating cortisol levels were measured at the end of the study. Corticosterone levels were the same in both the control and the stress group, suggesting that the mice did not respond differently to stress (Fig. 4*G*). The mean levels in all groups were elevated compared with what is found in normal, nonstressed mice, typically <46 ng/mL and stressed levels are typically >100 ng/mL (31–34). To measure food intake, the mice were singly housed throughout the study, which could increase stress in all groups. In addition, the corticosterone levels were measured in blood collected 36 h after the last stress induction. This collection would be after the expected peak corticosterone levels (35), which may explain the smaller differences in the control compared with the stress groups, resulting in no differences between the groups. Body weight increased in the nonstressed control group during this 1-wk period. In contrast, their littermates which were subjected to stress lost weight. These effects were not significantly different between genotypes (Fig. 4*H*). We also assessed the effects of stress on body composition. The effect on fat mass was proportionally greater, resulting in significant decreases in percent body fat (Fig. 4*I*). Notably, both total mass and fat mass were reduced significantly more in response to stress in the Moo1V^BTBR/BTBR^ genotype compared with Moo1V^B6/B6^ mice (Fig. 4, *J* and *K*). Stress significantly reduced fat mass (Fig. 4*J*) and lean mass (Fig. 4*L*) in both genotypes. In the nonstressed control mice, the change in these parameters did not differ between genotypes. This suggests that stress affects each genotype differently. Food intake was significantly reduced in Moo1V^BTBR/BTBR^ mice compared with Moo1V^B6/B6^ mice (Fig. 4*M*). The difference between genotypes was similar in both control and stress groups. As we expected with a decrease in adiposity in the stressed mice, we found that under stress, the Moo1V mice had improved glucose tolerance (Fig. 4, *N*–*P*).

Our observations that the *Moo1* phenotype is modulated by housing environment, and body weight analysis was performed in CDM *room 2* before we obtained the *Pdk1* and *Itga6* mutant mice, we repeated the Moo1-V strain body weight analysis in the newly renovated facility, CDM *room 3*. This analysis was performed in the same location and simultaneously with *Pdk1* and *Itga6* mutant mice. In this housing location, although the effects of *Moo1a* on body weight were blunted, body fat was still reduced in mice with the Moo1V^B6/B6^ mice (Supplemental Fig. S5).

### Effects of Reduced *Pdk1* on Body Weight

*Pdk1* encodes pyruvate dehydrogenase kinase 1. There are four kinases in this family that phosphorylate and inhibit pyruvate dehydrogenase (PDH), blocking the conversion of pyruvate to acetyl-CoA for entry into the Krebs cycle for glucose metabolism (36, 37). Reduced PDK1 is expected to increase acetyl-CoA formation. Acetyl-CoA is a substrate for lipogenesis and its activation could thereby promote fat storage. Thus, *Pdk1* is both a positional and functional candidate gene, with a reduction in PDK1 activity expected to promote obesity. We tested this hypothesis using global *Pdk1* KO mice and validated these KO mice using qPCR and western blots (Supplemental Fig. S6).

**Figure 5.**
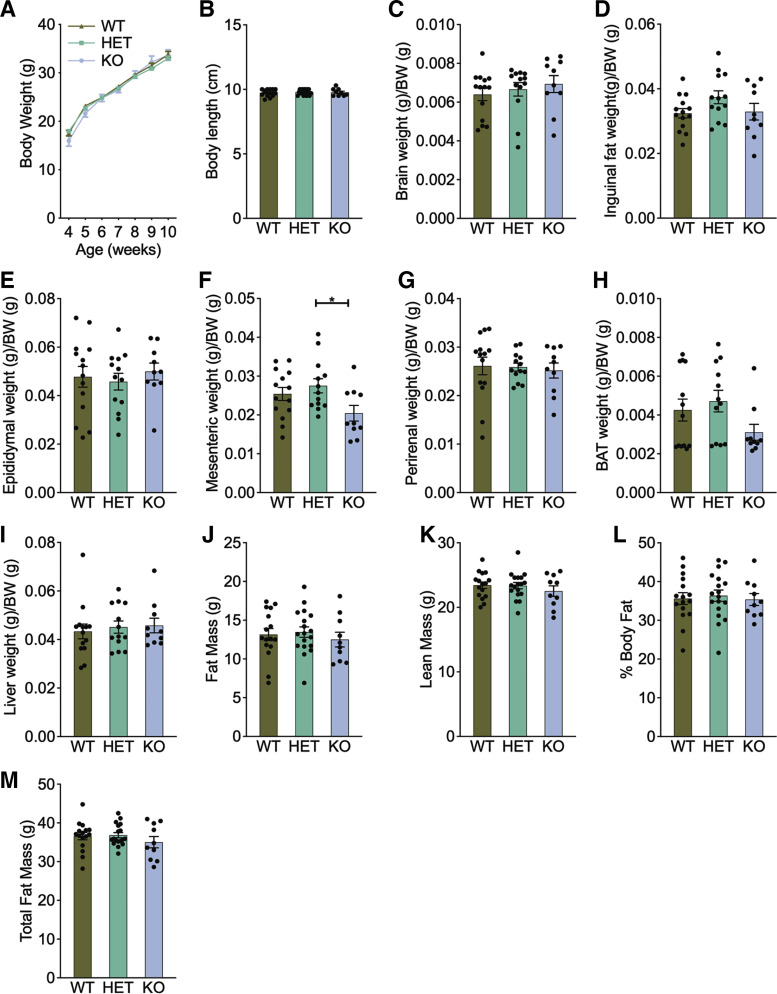
Effect of *Pdk1* on body weight, composition, and tissue weight. *A*: *Pdk1* knockout (KO), heterozygous (HET), and wildtype (WT) littermate mice were assessed weekly for body weight from 4 to 10 wk of age. *n* = 6–14. At 16 wk, *Pdk1* mice were euthanized and were measured for their body length (*B*), brain weight (*C*), inguinal fat (*D*), epididymal fat (*E*), mesenteric fat (*F*), perirenal fat (*G*), brown adipose tissue (BAT) (*H*), and liver weight (*I*). KO *n* = 9, HET *n* = 18, WT *n* = 16. Body composition analysis in 16-wk-old mice measuring fat mass (*J*), lean mass (*K*), % body fat (*L*), and total mass (*M*) did not differ between KO, HET, and WT mice. KO *n* = 10, HET *n* = 18, WT *n* = 16. Data are expressed as means ± SE and were analyzed using one-way ANOVA (*P* value shown). Tukey’s pairwise comparisons between groups that were significantly different are shown (*P < 0.05).

We first analyzed the role of *Pdk1* KO in body weight and measured weekly body weights to 10 wk of age. Growth curves from *Pdk1* KO mice were not significantly different from those of their HET or wildtype (WT) littermate controls (Fig. 5*A*). A lack of difference in total body weight does not necessarily indicate that there are no changes in adiposity. We measured adiposity as well. We did not detect a significant difference in body composition measured by DEXA in the KO mice compared with littermate HET and WT controls (Fig. 5*A*). We also measured body length to identify if there were differences but found no significant differences (Fig. 5*B*). We also found no significant differences in brain, inguinal, and epididymal fat weight (Fig. 5, *C–E*). However, we found decreases in mesenteric fat in *Pdk1* KO mice when compared with HET mice (Fig. 5*F*). This contrasts with our expectation from the congenic mice, where the genotype with reduced *Pdk1* expression had increased adiposity. We then assessed the weights of perirenal and brown adipose tissue and observed no differences (Fig. 5, *G* and *H*). The liver is a highly metabolic tissue, which may accumulate fat causing hepatic steatosis. However, when we weighed livers, we found no differences in the Pdk1 KO mice (Fig. 5*I*). Together, these studies, under the described experimental conditions, were unable to confirm that loss of Pdk1 mediates the effect of the *Moo1* locus on obesity (Fig. 5, *J–M*).

### Effects of Reduced *Pdk1* on Lipid Metabolism

**Figure 6.**
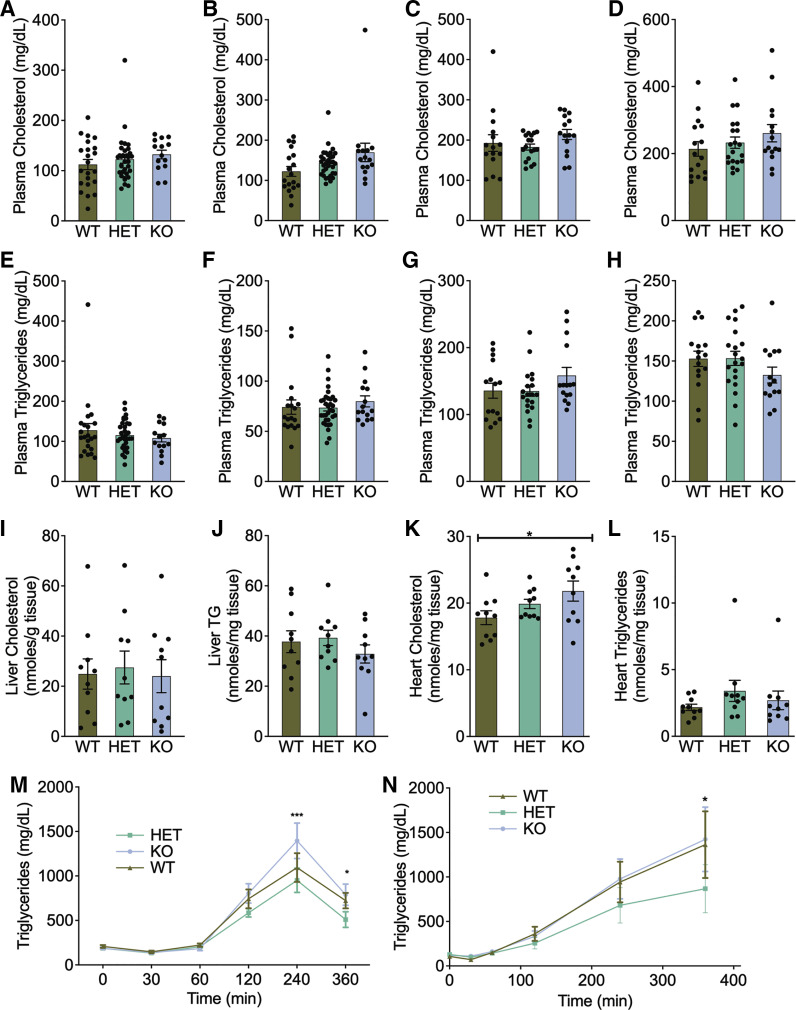
Lipid levels in fasted and nonfasted *Pdk1* mice. Plasma cholesterol levels were measured in nonfasted mice at 6 wk (*A*), fasted mice at 8 wk (*B*), nonfasted mice at 16 wk (*C*), and fasted mice at 16 wk (*D*). Plasma triglyceride levels were measured in nonfasted mice at 6 weeks (*E*), fasted mice at 8 wk (*F*), nonfasted mice at 16 wk (*G*), and fasted mice at 16 weeks (*H*). *n* = 14–30. Liver cholesterol (*I*) and triglyceride (*J*) and heart cholesterol (*K*) and triglyceride (*L*) were measured in fasted *Pdk1* knockout (KO), heterozygous (HET), and wildtype (WT) mice at 16 wk. *n* = 10 in each group. *M*: oral lipid tolerance tests were performed on 14-wk-old *Pdk1* KO, HET, and WT mice. *n* = 12–20. *N*: triglyceride appearance in the plasma during fasting, following lipase inhibition with poloxamer 407 was measured in 12 wk old mice. *n* = 12–18. Data are expressed as means ± SE and were analyzed using repeated-measures ANOVA with genotype and time as factors (*P* value shown). Tukey’s pairwise comparisons between groups at each time point that were significantly different are shown (*P < 0.05, ***P < 0.001, for HET vs. KO).

Studies have shown that reducing other isoforms of PDKs may contribute to changes in lipid metabolism and may differ in fed and fasted states (38–40). However, the in vivo effects of *Pdk1* loss on lipid metabolism were not known. We performed the analysis of lipid clearance and triglyceride synthesis in *Pdk1* KO mice to assess lipid metabolism. We analyzed the effects of reduced *Pdk1* (Supplemental Fig. S6) on metabolites in both fasted and nonfasted states. Plasma and liver triglyceride and cholesterol levels did not significantly differ in mice lacking *Pdk1* (Fig. 6, *A–J*). Although heart cholesterol levels were significantly increased (Fig. 6*K*), triglyceride levels were unaffected (Fig. 6*L*). To further our understanding of the change in lipids in *Pdk1* KO mice, we performed an oral lipid tolerance test. Although plasma triglyceride levels in these KO were not significantly different (Fig. 6*M*), KO mice were found to have decreased lipid clearance, increased triglyceride levels compared with the HET mice at 4- and 6-h post oral lipid challenge (Fig. 6*N*). The triglyceride appearance can be an indicator of lipid digestion and absorption from the intestine, and was similar in all genotypes. Changes in plasma lipid levels could indicate that there may be changes in lipid synthesis and secretion by the liver. To test this, we measured triglyceride secretion in *Pdk1* KO mice using a standard assay (29). This assay assesses lipid production during fasting by blocking triglyceride clearance and uptake using poloxamer 407. The WT mice were similar to the KO mice, which is expected because this cohort of *Pdk1* KO mice did not have differences in plasma triglyceride levels (Fig. 6*N*). Interestingly, the HET mice had reduced triglyceride secretion compared with the KO and WT mice throughout; however, the data were only statistically significant at 6-h post-injection of poloxamer 407 (Fig. 6*N*). Collectively, these observations reveal a potential novel role for PDK1 protein in cardiac cholesterol accumulation.

### Effects of Reduced *Itga6* on Body Weight

Integrins are a family of cell adhesion molecules comprised of a heterodimer of an α and β subunit. Integrin α 6 (ITGA6) binds with either the β1 or β4 subunits to form heterodimers, which are involved in cell-to-cell adhesion and cell adhesion to the extracellular matrix (41). ITGA6 is known to affect neuronal development and patterning (42, 43). Global *Itga6* knockout mice show disrupted neurulation and axial development (44). Altered neuronal development in areas controlling food intake and/or affecting energy expenditure could theoretically lead to obesity. Thus, *Itga6* is also both a functional and positional candidate gene to mediate the obesity effects at the *Moo1* locus. We hypothesized that reduced ITGA6 activity would promote obesity and tested this hypothesis using mice with partial deletion of *Itga6*. Moo1V^BTBR/BTBR^ mice were heavier than the Moo1V^B6/B6^ mice and the Moo1V^BTBR/BTBR^ had a 50% reduced *Itga6* gene expression. Thus, we hypothesized the *Itga6* HET mice would have increased body weight.

**Figure 7.**
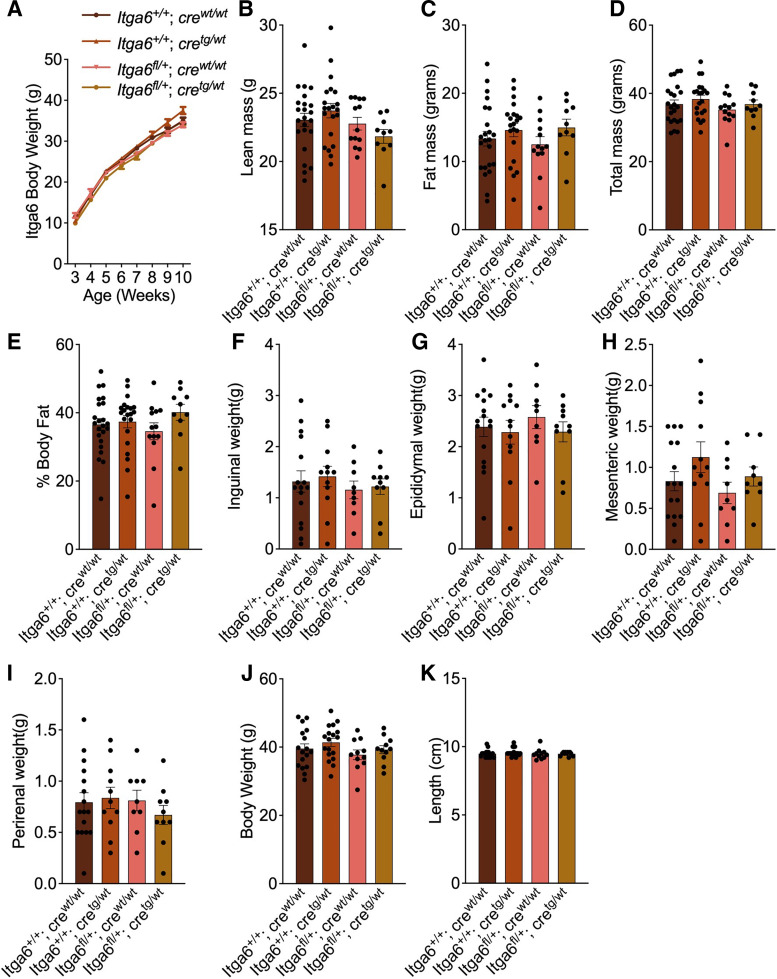
Effect of *Itga6* on body weight, composition, and tissue weight. *A*: growth curves of heterozygous knockout *Itga6*^fl/wt^;Cre^tg/wt^ (HET KO), *Itga6*^wt/wt^;Cre^tg/wt^ (CRE), flox positive *Itga6*^fl/fl^;Cre^wt/wt^ (FLOX), and wildtype *Itga6*^wt/wt^;Cre^wt/wt^ (WT) mice, graphed as mean weekly body weight until 10 wk of age, as assessed in our congenic studies; *n* = 21–35. Lean mass (*B*), fat mass (*C*), total fat mass (*D*), and % body fat (*E*), Inguinal (*F*), epididymal (*G*), mesenteric (*H*), and perirenal fat pad weights (*I*), body weight (*J*), and body length (*K*) were measured in 12-wk-old *Itga6* KO and control animals. Data are expressed as means ± SE and were analyzed using one-way ANOVA (*P* value shown).

We used heterozygous knockout mice in our studies because a homozygous KO *Itga6* mouse is not viable, dying shortly after birth^261^. To determine if reduced Itga6 affects body weight, we measured growth curves as in our previous studies. HET KO mice growth curves were not significantly different compared with their WT, FLOX, or CRE littermates. To ensure differences in adiposity were not missed, e.g., due to differences in body size (length) or changes in only specific fat pads, we also directly assessed body fat. In contrast to our expectations, we did not detect differences in body fat measured by DEXA, nor differences in the individual fat pad weights (Fig. 7, *A–K*). These data potentially identify that a global 50% reduction of *Itga6* alone is insufficient to modify body weight, at least using this Cre model under the conditions we studied. An important caveat of these studies is that at least in the liver in our studies, we were unable to detect a significant reduction of *Itga6* in our HET KO mice compared with their controls (Supplemental Fig. S7).

## DISCUSSION

The goal of the present study was to elucidate the causal genes that contribute to obesity at the Moo1 QTL. Unfortunately, despite conducting in vivo genetic loss-of-function studies on the two leading candidate genes, *Pdk1* and *Itga6*, we were unable to unambiguously point to a single causal gene that mediates the obesity phenotype. Nevertheless, a QTL affecting body weight was localized to the region contained in the Moo1V strain (*Moo1a*). Our inability to identify the mechanism by which this locus affects obesity could have many explanations. The effects of this locus on obesity appear to be susceptible to modulation by the environment (i.e., stress and diet), which likely complicate our efforts to identify the causal gene. It is also possible that both genes must be reduced to see the effects on obesity. Another possibility is that a SNP in the *Moo1* region may have long-range interaction or gene × gene interactions within the locus that affect a gene or genes outside the locus. There is precedent for variants affecting genes even 1 Mb away (45, 46). One of the expressed sequence tags (ESTs), perhaps a directly adjacent gene, *Rapgef4*, or an unknown element causes phenotype; however, there are no known miRNAs in the region. More studies will be needed to elucidate this mechanism. There were coding sequence changes in *Pdk1* and *Itga6* (Table 1) that were not predicted using biochemical models to have any function. However, the effects of these sequence changes on protein function were not tested. Thus, there is a possibility that these sequence changes leading to a gain or loss of function or other ESTs in the Moo1V strain region may alter obesity, instead of reduced *Pdk1* or reduced *Itga6* expression.

Moo1 QTL has multiple loci: one in the region of Moo1V (*Moo1a*), at least one in the region of the Moo1U strain, that is most likely located within the Moo1G strain but not within the Moo1L strain (*Moo1b*). This region extends from rs13476570 (top of gray zone for Moo1G) to rs27982530 (top of known B6 region in the Moo1L strain). This ∼1.8 Mb region where *Moo1b* is likely located is largely identical by descent between B6 and BTBR mice, with only 111 variants (of 55349 known SNPs and indels) listed as polymorphic between these two strains (retrieved from https://phenome.jax.org/snp/retrievals), none of which are annotated to affect protein-coding regions or splice sites. This is markedly in contrast to the 3416 (of 9593 known) variants retrieved from the 316 kb region containing *Moo1a* that differ between B6 and BTBR. Interestingly, *Moo1b* contains part of *Rapgef4*, also known as *Epac2*, which regulates cAMP signaling and is another potential candidate gene. Given the larger effect size, we focused on *Moo1a*. The Moo1T strain, which contains *Rapgef4*, does not have a clear obesity phenotype. It is possible that SNPs in Moo1T and not in Moo1V affect this gene causing effect on body weight in opposite direction.

In the current studies, we found that the housing environment modified the obesity phenotype of *Moo1*. We found potential suggestive evidence that stress may differentially affect the alleles of *Moo1a*. An increase in stress would counteract the increased adiposity associated with the inheritance of BTBR alleles in this region. Preliminary findings also suggested that the orexin pathway may be involved in the interaction between stress and chromosome 2 genotype. Interestingly, *Pdk1* and *Itga6* have been implicated in the orexin pathway and response to restraint stress, respectively (47–51), providing additional support for the findings of these studies.

The significant positive finding from this work was that reduced *Pdk1* affects cholesterol changes in the heart. PDK1 inhibitors have been proposed as potential cancer drugs (52, 53) and consistent with our findings, the PDK inhibitor AZ10219759 was reported to cause lipid accumulation in the heart with subsequent necrosis and atrial tissue degeneration (54). However, the mechanism by which PDK contributes to lipid accumulation is unknown (55). *Pdk1* may have metabolic effects in the heart that should be considered when using *Pdk1* inhibitors as a potential therapeutic. Further studies are needed to understand the role of PDK in cardiac lipid accumulation, ideally using cardiomyocyte-specific knockout mice.

Some of the limitations of these studies were that we were unable to replicate body weight data from one location to another. There have been known challenges where scientists are unable to replicate results when studies are repeated in different laboratories (56, 57). In our studies, we used only high-fat diets to be consistent throughout the genetic studies, but a chow diet was not used. Differences in methodology, environment, diet, genes or genetic background, microbiome changes, seasonal changes, sample size, and statistical power, can all affect the results of an experiment (58). In our studies, there have been differences in environment that were beyond our control, including changing mouse housing locations due to construction and stress from the surrounding environment that was beyond our control (e.g., construction) that likely affected our ability to repeat the same measurements and results. We were unable to detect a role for a HET knockout of *Itga6* on increasing body weight, but we are unable to make firm conclusions about *Itga6* because *Itga6* expression in livers collected at the end of the study was not significantly reduced in HET knockout mice. However, *Itga6* is an essential gene, whereby global knockout of *Itga6* is lethal. Homozygous *Itga6* knockout mice die shortly after birth due to extensive skin blistering (24). Thus, *Itga6* is an important gene and should not be disregarded.

Although we were unable to demonstrate evidence of the contribution of *Pdk1* and *Itag6* genes on body weight changes, there has been evidence of associations of this genetic region that are potentially associated with metabolic phenotypes. There is an association between *Pdk1* and various fat mass traits (59). SNPs near *Pdk1* and *Itga6* are potentially associated with many metabolic phenotypes in the human population, such as BMI, fasting glucose, type 2 diabetes, total cholesterol, and triglycerides (60). These SNPs are located between genes, so it is not clear which gene in the region they affect. However, this provides support that this region of the genome could affect metabolic traits in humans. Additional support for *Itga6* comes from genetic studies of other inbred strains (A/J and SM/J) to identify genes involved in impaired glucose metabolism, which identified a congenic strain affecting these traits encompassing *Itga6* but not *Pdk1* (61). The studies conducted in this paper highlight the importance, and the challenge of physiological studies in mice. It is important to note when there are noise windows, construction or facility changes during experiments. There are changes within the facility and environmental factors are hard to control in mouse studies, but may have significant and profound effects on phenotypes and the ability to reproduce phenotypes. Despite these challenges, these studies showed that PDK1 may be important for cholesterol changes in the heart.

## DATA AVAILABILITY

Data will be made available upon reasonable request.

## SUPPLEMENTAL DATA

10.6084/m9.figshare.20723956 Supplemental Tables S1 and S2 and Figs. S1–S7: https://doi.org/10.6084/m9.figshare.20723956.

## GRANTS

This work was supported by a Tier 2 Canada Research Chair Award and a Canadian Institutes of Health Research Grant (to S. M. Clee).

## DISCLOSURES

No conflicts of interest, financial or otherwise, are declared by the authors.

## AUTHOR CONTRIBUTIONS

C.L.K.L. and S.M.C. conceived and designed research; C.L.K.L., S.K., L.I., and X.H. performed experiments; C.L.K.L., S.K., and L.I. analyzed data; C.L.K.L., V.V., and S.M.C. interpreted results of experiments; C.L.K.L. and M.G.A. prepared figures; C.L.K.L. drafted manuscript; C.L.K.L., S.K., M.G.A., J.D.J., and S.M.C. edited and revised manuscript; C.L.K.L., S.K., M.G.A., L.I., X.H., V.V., and J.D.J. approved final version of manuscript.
